# Paralysie néonatal unilatérale du nerf radial

**DOI:** 10.11604/pamj.2015.21.283.7276

**Published:** 2015-08-13

**Authors:** Halima Benemmane, Fouzia Hali, Farida Marnissi, Hakima Benchikhi

**Affiliations:** 1 Service de Dermatologie, CHU Ibn Rochd, Casablanca, Maroc; 2 Service d'Anatomopathologie, CHU Ibn Rochd, Casablanca, Maroc

**Keywords:** Nerf radial, cytostéatonécrose, néonatale, paralysie, radial nerve, cytosteatonecrosis, neonatal, paralysis

## Abstract

La paralysie néonatale unilatérale du nerf radial est rare, son diagnostic est essentiellement clinique, elle peut-être diagnostiquée à tort en tant que paralysie du plexus brachial. Nous rapportons un cas clinique. A l'examen clinique du nouveau-né; l'extension du poignet, du pouce et des articulations métacarpo-phalangiennes était impossible, alors qu'il y avait une conservation de la prono-supination et la flexion du poignet et des mouvements de l’épaule et du coude. Le diagnostic de la paralysie du plexus brachial était écarté cliniquement devant la mobilisation active de l’épaule et la flexion du coude. Notre patient a bénéficié de kinésithérapie pour éviter l'apparition d'attitudes vicieuses et d'amyotrophie. L'extension active du poignet était obtenue après deux mois.

## Introduction

La paralysie néonatale isolée du nerf radial est rare, son diagnostic est essentiellement clinique, elle peut-être diagnostiquée à tort en tant que paralysie du plexus brachial [1–3]. Nous en rapportons un cas associé à une cytostéatonécrose néonatale (CSN), traité par physiothérapie.

## Patient et observation

**Figure 1:**
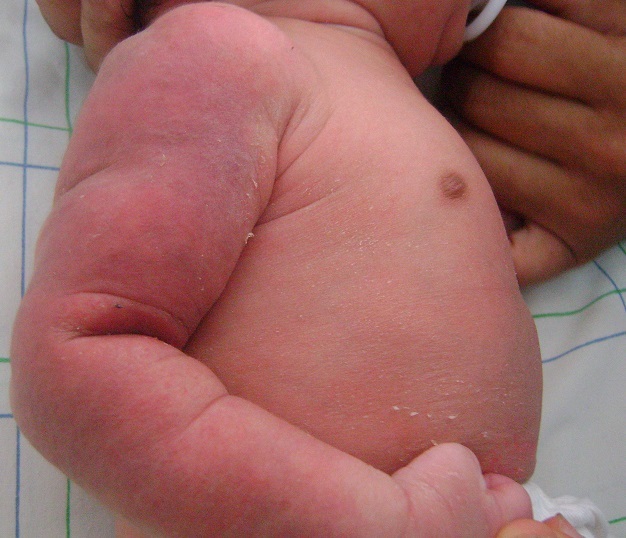
Placard sous-cutané rouge violacé douloureux du bras droit à J3

**Figure 2:**
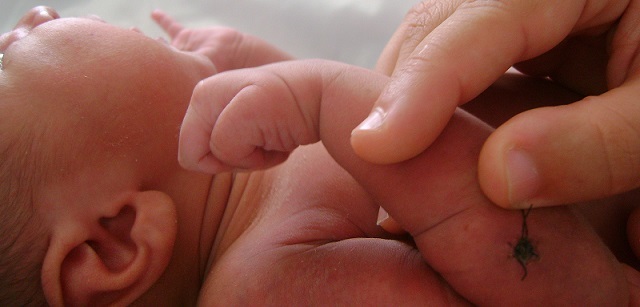
L'extension du poignet, du pouce et des articulations métacarpo-phalangiennes était impossible

**Figure 3:**
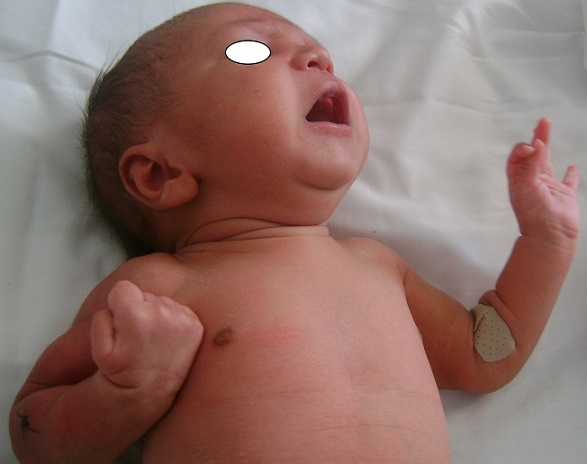
Les mouvements de l’épaule, du coude, la prono-supination et la flexion du poignet étaient possibles

Un nouveau-né de sexe masculin était issu d'une grossesse menée à terme. Sa mère était une primigeste à bassin limite, elle avait un accouchement dystocique fait par voie basse après un long travail. A la naissance, le nouveau-né pesait 3100 g, son score d'Apgar était 5/10, son périmètre crânien était 34 cm, sa taille était 47cm. Il était exclusivement allaité au sein. Au troisième jour de sa vie, il avait présenté des nodules sous-cutanés rouges violacés douloureux au niveau du bras droit évoluant vers l'extension à tout le membre supérieur droit (Figure 1). L'examen du membre supérieur droit avait montré que l'extension du poignet, du pouce et des articulations métacarpo-phalangiennes était impossible (Figure 2), avec conservation de la prono-supination et la flexion du poignet des mouvements de l’épaule et du coude homolatéraux (Figure 3). La radiographie du bras avait éliminé une fracture de l'humérus. L’électromyogramme (EMG) fait à J8 avait objectivé une dénervation isolée du nerf radial. La biopsie cutanée avait montré un granulome lipophagique et une nécrose lipocytaire. La calcémie, la glycémie et le bilan lipidique étaient normaux. L’échographie rénale était normale. Le nouveau-né avait bénéficié de séances de physiothérapie à type de mouvements passifs avec mise en place d'une attelle statique pendant deux mois. L'extension du poignet était obtenue après deux mois.

## Discussion

La limitation unilatérale des mouvements du membre supérieur chez un nouveau-né peut-être due soit à une atteinte neurologique ou une pseudoparalysie (fracture de la clavicule ou de l'humérus [4], une arhtrite septique ou une ostéomyélite) [2]. La paralysie isolée du nerf radial est caractérisée par l'abolition de l'extension du poignet, du pouce et des articulations métacarpo-phalangiennes, avec conservation de la rotation externe de l’épaule et des mouvements du coude. Le problème du diagnostic différentiel se pose avec la paralysie du plexus brachial surtout dans ses formes légères, où il y'a une paralysie de l’épaule, une réduction de la flexion du coude et une limitation de la rotation externe du membre supérieur. La paralysie unilatérale néonatale est fort probablement secondaire à une pression prolongée, exercée par l'anneau pelvien sur la partie inférieure du bras, soit in utero ou durant la délivrance [1]. La compression in utero est mise en évidence par la dénervation active du muscle objectivée par l'EMG la première semaine de vie [5]. Hyman et al trouvent plutôt que les lésions surviennent au cours du travail [6]. Coppotelli avait noté la présence d'une marque de pression sur le bras d'un nouveau né dont la biopsie avait montré une cytostéatonécrose traumatique en faveur d'un processus prolongé [7]. Le travail prolongé et l'accouchement dystocique présentent des facteurs de risque de la (CSN) [8–10]. Ce diagnostic a été confirmé histologiquement par la présence de granulome lipophagique et de nécrose lipocytaire à la biopsie cutanée. Une étude qui porte sur 25 nouveau-nés qui avaient une paralysie isolée du nerf radial a trouvé que 68% parmi eux avaient une cytostéatonécrose [1]. La récupération du nerf radial est obtenue dans une période allant d'une à six semaines [1], dans l’étude de Alsubhi et al 72% des nouveaux-né ont récupéré après deux mois, néanmoins tous les patients ont fini par récupérer. Notre patient a bénéficié de physiothérapie afin d’éviter l'apparition d'attitude vicieuse et d'amyotrophie, plusieurs cas de paralysie néonatale isolée du nerf radial ont récupéré sans physiothérapie [1], dont l'effet est difficile à évaluer [1].

## Conclusion

La paralysie néonatale isolée du nerf radial est de pronostic favorable. Il faut la différencier de la paralysie du plexus brachial dont le pronostic est péjoratif.
